# Complete Genome Sequence of Bovine Viral Diarrhea Virus-1 Strain Egy/Ismailia/2014, Subtype 1b

**DOI:** 10.1128/genomeA.01518-15

**Published:** 2015-12-23

**Authors:** Mohamed A. Soltan, Rebecca P. Wilkes, Mohamed N. Elsheery, Mahmoud M. Elhaig, Matthew C. Riley, Melissa A. Kennedy

**Affiliations:** a Department of Veterinary Medicine, Faculty of Veterinary Medicine, Infectious Diseases Division, Suez Canal University, Ismailia, Egypt; b Department of Biomedical and Diagnostic Sciences, College of Veterinary Medicine, University of Tennessee, Knoxville, Tennessee, USA; c Veterinary Diagnostic and Investigational Laboratory, College of Veterinary Medicine, University of Georgia, Tifton, Georgia, USA; d Medical Service Corps, United States Army, USA

## Abstract

Here, we report the complete genome sequence of bovine viral diarrhea virus-1b (BVDV-1b), strain Egy/Ismailia/2014. The virus genome is composed of 12,217 nucleotides organized as one open reading frame encoding 3,898 amino acids. This report will assist efforts in diagnostics, studying molecular epidemiology, and control of BVDV in Egypt.

## GENOME ANNOUNCEMENT

Bovine viral diarrhea virus (BVDV) belongs to the genus *Pestivirus*, family *Flaviviradae*. It is a single-stranded RNA virus, with a genome length of about 12.5 kb organized as an open reading frame flanked by 5′ and 3′ untranslated regions (UTR) (1). BVDV is classified according to the biological activity in cell culture into cytopathic and noncytopathic biotypes (2). Furthermore, it is classified according to nucleotide sequence analysis of the 5′ UTR, N-terminal protease region (N^pro^) and E2 glycoprotein into three main species; BVDV-1, BVDV-2, and BVDV-3 (Hobi-like virus). BVDV-1 contains at least 20 subtypes and BVDV-2 contains 3 subtypes (3–5).

BVDV strain Egy/Ismailia/2014 was detected in an outbreak of BVDV in newborn calves under 2 months of age on a dairy cattle farm in Ismailia province. The detected strain was previously subtyped as BVDV-1b by nucleotide sequence analysis of the 5′ UTR, N^pro^ region and E2 glycoprotein (6). For the whole-genome sequencing, the nucleotide sequences of the 5′ UTR, N^pro^ region, and E2 glycoprotein were aligned with previously published genomes of BVDV-1b and 20 overlapping primers were designed from closely related genomes.

The assembly of all PCR products revealed amplification of a total of 12,217 bp of strain Egy/Ismailia/2014. The genome is composed of one open reading frame encoding 3,898 amino acids flanked by a 5′ UTR and 3′ UTR. The nucleotide and deduced amino acids sequences of the characterized strain were aligned with other published BVDV-1b strains, the nucleotide and amino acid homology ranged from 89.4% to 94.2% and 91.3% to 96.8%, respectively. The E2 glycoprotein was highly divergent, with nucleotide and amino acid homology ranging from 81.3% to 93.6% and 85.3% to 93.6%, respectively. Furthermore, the nonstructural glycoprotein 3 (NS3) was highly conserved, with nucleotide and amino acid homology ranging from 90.9% to 95.1% and 98% to 99.4%, respectively.

In this study, the BVDV genome was amplified directly from blood samples to avoid any source of BVDV contamination resulting from fetal bovine serum used in culture media and any mutations originating from the adaptation and replication of BVDV in cell lines. This is the first study to describe the complete genome sequence of BVDV from an Egyptian cattle population. This sequence gives valuable molecular data to trace the source of viral infection, mode of transmission, and vaccine selection.

### Nucleotide sequence accession number.

The complete genome sequence of BVDV Egy/Ismailia/2014 strain has been deposited in GenBank under the accession number KR029825.
